# *Cyclosorus terminans* Extract Ameliorates Insulin Resistance and Non-Alcoholic Fatty Liver Disease (NAFLD) in High-Fat Diet (HFD)-Induced Obese Rats

**DOI:** 10.3390/nu14224895

**Published:** 2022-11-19

**Authors:** Sujinda Songtrai, Wasana Pratchayasakul, Busarin Arunsak, Titikorn Chunchai, Aphisek Kongkaew, Nipon Chattipakorn, Siriporn C. Chattipakorn, Sireewan Kaewsuwan

**Affiliations:** 1 Department of Pharmacognosy and Pharmaceutical Botany, Faculty of Pharmaceutical Sciences, Prince of Songkla University, Songkhla 90110, Thailand; 2 Phytomedicine and Pharmaceutical Biotechnology Excellence Center, Faculty of Pharmaceutical Sciences, Prince of Songkla University, Songkhla 90110, Thailand; 3 Neurophysiology Unit, Cardiac Electrophysiology Research and Training Center, Faculty of Medicine, Chiang Mai University, Chiang Mai 50200, Thailand; 4 Cardiac Electrophysiology Unit, Department of Physiology, Faculty of Medicine, Chiang Mai University, Chiang Mai 50200, Thailand; 5 Center of Excellence in Cardiac Electrophysiology Research, Chiang Mai University, Chiang Mai 50200, Thailand; 6 Research Administration Section, Faculty of Medicine, Chiang Mai University, Chiang Mai 50200, Thailand; 7 Department of Oral Biology and Diagnostic Sciences, Faculty of Dentistry, Chiang Mai University, Chiang Mai 50200, Thailand

**Keywords:** *Cyclosorus terminans*, anti-diabetes, insulin resistance, obesity, HOMA-IR, fatty liver

## Abstract

Interruptins A and B exhibited anti-diabetic, anti-inflammatory, and anti-oxidative effects. This study aimed to investigate the therapeutic ability of extract enriched by interruptins A and B (EEI) from an edible fern *Cyclosorus terminans* on insulin resistance and non-alcoholic fatty liver disease (NAFLD) in a high-fat diet (HFD)-induced obese rats and elucidate their possible mechanisms. HFD-induced obese rats were treated with EEI for 2 weeks. Real-time polymerase chain reaction (PCR) was used to examine the molecular basis. We found that EEI supplementation significantly attenuated body and liver weight gain, glucose intolerance, and insulin resistance. Concurrently, EEI increased liver and soleus muscle glycogen storage and serum high-density lipoprotein (HDL) levels. EEI also attenuated NAFLD, as indicated by improving liver function. These effects were associated with enhanced expression of insulin signaling genes (*Slc2a2, Slc2a4, Irs1* and *Irs2*) along with diminished expression of inflammatory genes (*Il6* and *Tnf*). Furthermore, EEI led to the suppression of lipogenesis genes, *Srebf1* and *Fasn*, together with an increase in fatty acid oxidation genes, *Ppara* and *Cpt2*, in the liver. These findings suggest that EEI could ameliorate HFD-induced insulin resistance and NAFLD via improving insulin signaling pathways, inflammatory response, lipogenesis, and fatty acid oxidation.

## 1. Introduction

The incidence of non-communicable diseases (NCDs) has been increasing at a rapid pace, becoming a serious problem to human health and quality of life. Diabetes mellitus (DM) is one of the most frequently reported NCDs, with a persistent rise in the prevalence rate. The total number of people with diabetes worldwide was approximately 463 million in 2019, and the number is estimated to rise to 700 million in 2045 (51%) [1]. DM is a complex metabolic disorder that occurs as a consequence of genetic, lifestyle, and food habit changes. The most significant clinical indicator of diabetes is persistent hyperglycemia which is caused by either the failure of the pancreas to secrete sufficient insulin or by decreased sensitivity of target organs to insulin which is known as insulin resistance [2,3].

The latter is one of the serious problems that not only leads to glucose intolerance but also induces hepatic lipid accumulation, which further results in nonalcoholic fatty liver disease (NAFLD) [4,5]. In principle, NAFLD refers to hepatic steatosis or the accumulation of fat in the liver in the absence of excessive alcohol consumption. The term NAFLD comprises a broad spectrum of liver diseases ranging from steatosis to non-alcoholic steatohepatitis (NASH), fibrosis, and cirrhosis. NAFLD is frequently noticed as a comorbidity in patients with Type 2 diabetes mellitus (T2DM) [6].

Presently, the use of oral hypoglycemic drugs is the primary means considered for hyperglycemia control [2]. Although anti-diabetic drugs can be lifesaving, relieve symptoms, and prevent long-term diabetic complications, however, sub-therapeutic effects still occur, and most of the drugs cause adverse effects that diminish the quality of life for patients [7]. Therefore, there is a tremendous need to search for novel effective, and safe agents for countering insulin resistance to prevent the occurrence or development of diabetes and NAFLD.

Thiazolidiones (TZDs), including ciglitazone, rosiglitazone, and pioglitazone, are a class of antidiabetic drugs that act as insulin sensitizers through binding with peroxisome proliferator-activated receptor-gamma (PPARG) [8]. Moreover, the modulation of PPARA has been implicated in correlating with NASH [9]. The administration of PPARA agonists has been shown to decrease hepatic triglyceride (TG) accumulation in high-fat diet (HFD)-induced obese mice and up-regulate β-oxidation enzymes in HFD-induced obese mice, thereby reducing hepatic TG in obese mice [10].

In addition, reactive oxygen species (ROS) are continuously generated during hyperglycemic conditions. The induction of oxidative stress by the overproduction of ROS leads to diabetic progression. Antioxidants are important compounds in scavenging free radicals and protecting the body from oxidative stress. Therefore, neutralizing free radicals with antioxidants is one of the treatments and prevention against diabetes development and complications [11,12].

Recently, in vitro, some studies demonstrated that interruptins A and B from an edible fern *Cyclosorus terminans* have strong antioxidant activity [13,14], which is a sign of encouraging anti-diabetic activity. Moreover, they have shown the potential therapeutic effects for anti-inflammatory and anti-diabetic [15,16,17]. However, in vivo anti-diabetic activity of *C. terminans* is hitherto unknown, although it has been reported that interruptin B isolated from *C. terminans* induced brown adipogenic differentiation of adipose-derived stem cells (ASCs) and stimulated glucose consumption in differentiated ASCs through the role as a dual PPARA and PPARG ligands [15]. Taken together, both interruptins A and B were currently found to facilitate glucose consumption in FL83B mouse hepatocytes cells via induction of PPARA and PPARG protein expression [16]. It would therefore be interesting to understand the pharmacological features of *C. terminans* regarding glucose and lipid metabolism. The current study proceeded to investigate the pharmacological property of *C. terminans* extract enriched by interruptins A and B (EEI) on insulin resistance and fatty liver disease of obese rats induced by long-term HFD.

## 2. Materials and Methods

### 2.1. Plant Material and Extract Preparation

Aerial parts of fern, *C. terminans*, were collected from Nakornsrithammarat province, Thailand. The plant was identified by Prof. Dr. Thaweesakdi Boonkerd (Chulalongkorn University, Thailand), and a voucher specimen (no. SKP 2080320001) was kept at the Faculty of Pharmaceutical Sciences, Prince of Songkla University, Thailand. The extraction method was described in the previous report [17]. Briefly, dried fern powder was extracted with *n*-hexane under reflux. The *n*-hexane extract was then enriched with the active compound, interruptins A and B, by partial purification with vacuum column chromatography. All fractions containing interruptins were collected and dried. EEI was evaluated for the amount of interruptins A and B by the HPLC method [13,18]. The result showed that EEI contained 24.4% weight by weight (*w*/*w*) interruptin A and 9.2% (*w*/*w*) interruptin B [17]. EEI was stored in the refrigerator at 4 °C until use.

### 2.2. Animal Models

The experiment was conducted with the approved procedure. All experimental procedures were approved by Laboratory Animal Center, Chiang Mai University Animal Care and Use Committee, Chiang Mai University (Permit number: 2562/RT-0007) and conformed to the Guide for the Care and Use of Laboratory Animals published by an Assessment and Accreditation of Laboratory Animal Care International (AAALAC) guidelines. Male Wistar rats (180–200 g; *n* = 50) were employed in the present experiment and obtained from Nomura, Bangkok, Thailand. All animals were acclimatized in a temperature-controlled condition (25 ± 0.5 °C) with a 12:12 h light-dark cycle. After 1 week of acclimatization, rats were separated into two main groups, a normal diet (ND; *n* = 10) group and a high-fat diet (HFD; *n* = 40) group. In the ND group, rats were received on a typical laboratory diet containing 19.77% E from fat, 51.99% E from carbohydrate, and 28.24% E from protein with an energy content estimated at 4.02 kcal/g (Mouse Feed Food No. 082, C.P. Company, Bangkok, Thailand). Rats in the HFD group were received on a diet containing 57.60% E from fat, 1.68% E from cholesterol, 14.27% E from carbohydrate, and 26.45% E from protein with an energy content estimated at 5.35 kcal/g to provide the obese-insulin resistant models. All diets contained a mineral and a combination of vitamins. Water, food intake and body weight were observed every week.

### 2.3. Experimental Design

After 12 weeks, each dietary group was further subdivided into different treatment groups (*n* = 10).

Group 1 (ND; control): ND rats received a vehicle.

Group 2 (HFD; negative control): HFD rats received a vehicle.

Group 3 (EEI100): HFD rats received 100 mg/kg/day of EEI.

Group 4 (EEI200): HFD rats received 200 mg/kg/day of EEI.

Group 5 (Pio20; positive control): HFD rats received 20 mg/kg/day of pioglitazone. EEI and pioglitazone were suspended in a vehicle consisting of tween 80: PEG 400: distilled water (1:4:5) [17]. Each group was administered treatment daily for 2 weeks by oral gavage. All rats were continuously given their diet after the separated group. The ND group received a standard diet, whereas the HFD group received HFD during the experimental period.

### 2.4. Homeostasis Model Assessment of Insulin Resistance (HOMA-IR)

Insulin resistance was evaluated by the Homeostasis model assessment of insulin resistance (HOMA-IR) as mathematically derived nonlinear equations explaining the level of insulin resistance [19]. HOMA-IR was calculated using plasma insulin and glucose according to the following equations:

HOMA-IR = ((fasting plasma insulin in μU/mL) × (fasting plasma glucose in mg/dL))/405

### 2.5. Oral Glucose Tolerance Test (OGTT)

In the 14th week, OGTT was performed. All animal groups had fasted for 12 h before OGTT was achieved, and 2 g/kg body weight of glucose was administered by oral gavage. Then blood samples were immediately collected from the tip of the tail at 0, 15, 30, 60, and 120 min. The blood samples were contained in sodium fluoride tubes and then centrifuged at 2500 rpm 4 °C for 10 min. Separated plasma was stored at −80 °C until it was analyzed by an automated biochemical analyzer (ARCHITECTPlus ci8200, Abbott, Chicago, IL, USA) with its commercial kit. The total area under the curve (AUC) of plasma glucose was estimated by using a trapezoidal method.

### 2.6. Biochemical Parameter Analysis

At the end of the experiment, after fasting animals for 6 h, blood samples were drawn from the cervical arteries of animals. The blood samples were collected in ethylenediaminetetraacetic acid (EDTA) anti-clotting tubes and assessed for plasma insulin content using the Rat/Mouse Insulin ELISA kits (Millipore, St. Charles, MO, USA). Sodium fluoride tubes and plain glass tubes were provided to collect blood samples for evaluating other biochemical parameters. Tube samples were centrifuged at 2500 rpm 4 °C for 10 min. Plasma and serum were separated and kept at −80 °C for further evaluation. Separated plasma from sodium fluoride tubes was measured for glucose, and separated serum from plain glass tubes was assessed for lipid and liver profiles, including total triglyceride, total cholesterol, high-density lipoprotein (HDL), low-density lipoprotein (LDL), albumin, total protein, total bilirubin, direct bilirubin, alanine aminotransferase (ALT), aspartate aminotransferase (AST), and alkaline phosphatase (ALP). All assays were carried out using commercial kits in an automated biochemical analyzer (ARCHITECTPlus ci8200, Abbott, Chicago, IL, USA).

### 2.7. Liver Triglyceride Determination

Liver samples (30 mg) were lysed with radioimmunoprecipitation assay (RIPA) buffer (Thermo Fisher Scientific, Rockford, IL, USA). Thereafter, the homogenates were centrifuged at 14,000 rpm for 10 min to obtain supernatant containing lipids. Extracted lipids were dissolved in 100% isopropyl alcohol, followed by vigorous shaking for 10 min. The suspensions were centrifuged at 14,000 rpm for 10 min [20]. The supernatant, including triglycerides, was measured using a commercial triglyceride assay (Triglycerides, Erba Mannheim, Brno, Czech Republic) according to the manufacturer’s recommendations. The values of triglycerides were expressed as mg/g of the harvested livers.

### 2.8. Glycogen Content

Livers and soleus muscles (20 mg) tissues were carried out in lysis buffer (RIPA) on ice. To inactivate enzymes, tissue solutions were boiled for 10 min and then centrifuged at 4 °C and 14,000 rpm for 10 min. Supernatants were treated with 95% ethanol at 40 °C for 4 h to precipitate glycogen. Samples were centrifuged at 4 °C and 14,000 rpm for 10 min and then discarded supernatant. Precipitates were dried, and the glycogen content was evaluated using the anthrone method [21]. Briefly, 0.2% anthrone (in 95% sulfuric acid) was put into the samples, followed by boiling for 15 min. The reaction was monitored with the blue chromophore. Glucose solution was used as standard, whereas distilled water was used as blank. After cooling the solutions to room temperature, the blue color was measured at 620 nm utilizing a microplate reader (BMG labtech, Ortenberg, Germany). The concentration of glycogen was estimated from the glucose standard curve and normalized against the weight of the extracted liver and soleus muscle.

### 2.9. Gene Expression Analysis

10 mg rat liver or soleus muscle was extracted for total RNA using an RNA extraction kit (FavorPrep^TM^ Blood/Cultured Cell Total RNA Purification Mini Kit, Favorgen Biotech, Ping-Tung, Taiwan) and transcribed to cDNA using the FIREScript RT cDNA Synthesis KIT (Solis Bio-Dyne, Tartu, Estonia) with oligo (dT) 18 primer following manufacturer’s instructions. For real-time PCR, samples of cDNA were triplicate analyzed using the 5× HOT FIREPol^®^ EvaGreen^®^ qPCR Mix Plus (Solis Bio-Dyne, Tartu, Estonia) on a QuantStudio 3 Real-Time PCR System (Applied Biosystems, Waltham, MA, USA). The levels of relative mRNA were analyzed and normalized to β-actin (*Actb*), used as an endogenous control gene. The final concentration of primers in the reaction was 250 nM, and their sequences are presented in Table 1. Thermal cycling for PCR was as follows: activation of 94 °C for 15 min, followed by 40 cycles of amplification at 94 °C for 15 s, 54–59 °C for 20 s, and 72 °C for 27 s.

### 2.10. Macroscopic and Microscopic Examination

After each rat was euthanized, its intact liver was removed, rinsed with normal saline solution, and dried on filter paper. The liver was weighed and then grossly examined. Ten mm samples were carried from the right lobes of the liver and cut into sections. Tissue sections were fixed by directly soaking in 10% formalin at room temperature for 24 h. The specimens were then dehydrated in ascending grades of ethanol. The specimens were immersed in xylene and paraffin-embedded, then sliced into 5 µm thick sections and stained with hematoxylin and eosin (H&E). The general histological structures of the treatment groups were compared with the control group. The histopathology of H&E liver sections was evaluated following a modified Kleiner scoring system [22]. Steatosis and hypertrophy characteristics were observed under a microscope at a 40–200× magnification with only the sheets of hepatocytes that preclude terminal hepatic venules and portal tracts. Inflammation was examined at a 100× magnification by counting the number of inflammatory foci per field (view size of 3.1 mm^2^). The score is based on the percentage of the total area into the subsequent categories: 0 (<5%), 1 (5–33%), 2 (34–66%), and 3 (>66%).

### 2.11. Statistical Analysis

The data are presented as mean ± standard error of the mean (SEM). The normal distribution of variables was analyzed using a 1-way analysis of variance (ANOVA) with SPSS software version 22. Significant differences between means were performed by the Least Significant Difference (LSD) test. The abnormal distribution data were performed by using the Kruskal–Wallis test, followed by the Dunn–Bonferroni post hoc test to determine the difference between groups. Statistical significance presented at *^, #^ *p* < 0.05 and **^, ##^ *p* < 0.01 levels.

## 3. Results

### 3.1. EEI Reduced Body Weight of HFD-Induced Obese Rats

**Figure 1:**
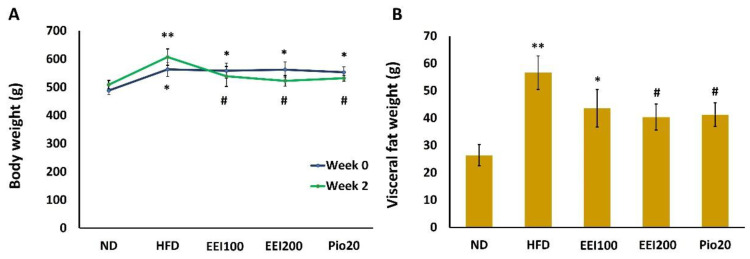
The effect of 2-week feeding EEI on body weight (**A**) and visceral fat weight (**B**) of rats. Results are presented as the means ± SEM (*n* = 10). Significant differences were presented as * *p* < 0.05 and ** *p* < 0.01 compared to the ND group, ^#^ *p* < 0.05 compared to the HFD group. ND = Normal diet-fed rats treated with vehicle; HFD = High fat diet-fed rats treated with vehicle; EEI100 = HFD-fed rats treated with 100 mg/kg/day of EEI; EEI200 = HFD-fed rats treated with 200 mg/kg/day of EEI; Pio = HFD-fed rats treated with 20 mg/kg/day of pioglitazone.

The experimental rats were fed with HFD for 3 months to induce insulin resistance and obesity and were simultaneously administered with EEI (100 and 200 mg/kg/day) or pioglitazone (20 mg/kg/day) for 2 weeks. All animals were weighed every week. After 12 weeks of the dietary period (week 0 of treatment), HFD-fed rats showed the characteristic of obesity as indicated by increased body weight compared to the ND group. After 2 weeks of treatment, the body weight of animals was substantially reduced in all EEI-treated groups compared to the HFD group. Likewise, the pioglitazone group did demonstrate decreased body weight compared to the HFD group (Figure 1A). Furthermore, HFD-fed rats also increased visceral fat weight when compared to ND-fed rats. Interestingly, 200 mg/kg/day of EEI and pioglitazone shared similar effects on the attenuation of visceral fat in HFD-fed rats (Figure 1B). Through these findings, EEI was confirmed to be effective in reducing body weight gain induced by HFD.

### 3.2. EEI Improved Insulin Resistance and Hyperglycemia in HFD-Induced Obese Rats

To investigate the insulin resistance ameliorating effect of EEI, we employed insulin-resistant-obese rats induced by HFD. As shown in Table 2, the plasma insulin level and HOMA-IR index of the HFD group were visibly higher than those in the ND group. These results indicate that HFD-fed rats developed insulin resistance. Interestingly, 200 mg/kg/day EEI supplementation significantly alleviated an increase in the plasma insulin level and HOMA-IR index when compared to the HFD group. A similar result was observed in the 20 mg/kg/day pioglitazone group. These results implied that EEI treatment reduced insulin resistance induced by HFD. In addition, the plasma glucose level in HFD-fed rats was significantly elevated when compared with the ND group. However, the plasma glucose levels were significantly lower in 100 and 200 mg/kg/day EEI-treated groups when compared with the HFD group (Table 2), suggesting that EEI could attenuate hyperglycemia caused by HFD consumption.

It was also found that the AUC of plasma glucose for OGTT of the HFD group was markedly higher than that of the ND group. These findings indicated that HFD consumption led to impaired glucose tolerance. The administration of 100 and 200 mg/kg/day EEI significantly decreased the AUC of glucose tolerance in a dose-dependent manner in HFD-fed rats compared to the HFD group (Table 2), which was similar to the corresponding effect of the standard drug pioglitazone. These results suggested that EEI supplementation improved the condition of impaired glucose tolerance induced by HFD consumption.

### 3.3. Effects of EEI on Serum Lipid Parameters and Liver Enzymes

Besides the effect of EEI on attenuating insulin resistance induced by HFD ingestion, we further examined whether EEI affects serum lipids after HFD feeding. We found that the levels of total cholesterol LDL level significantly increased, but HDL levels became lower, and triglyceride levels did not change in the HFD group when compared with the ND group. It was observed that administration of EEI did not alter the values of serum lipid profiles except HDL level. Our findings indicated a significant increase in HDL level (30.6%) in the 200 mg/kg/day EEI-treated group, whereas the 20 mg/kg/day pioglitazone group demonstrated no significant change in HDL levels when compared with that of the HFD group. Nonetheless, pioglitazone significantly reduced levels of triglyceride and total cholesterol (Table 3).

Furthermore, the levels of AST, ALT, and ALP were significantly increased in HFD-fed rats when compared to ND-fed rats. Interestingly, the administration of 200 mg/kg EEI and 20 mg/kg pioglitazone equally attenuated the increase of these liver enzymes in HFD-fed rats.

These results indicated that EEI administration did not affect the profile of potentially harmful lipids but elevated the levels of the protective HDL in the bloodstream. More importantly, treatment with EEI ameliorated liver dysfunction induced by HFD.

### 3.4. EEI Enhanced the Hepatic and Soleus Muscle Glycogen Content in HFD-Induced Obese Rats

**Figure 2:**
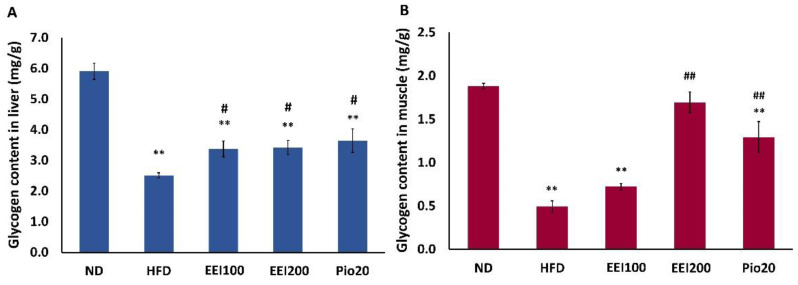
The effect of 2-week feeding EEI on glycogen content in the liver (**A**) and soleus muscle (**B**). Results are presented as the means ± SEM (*n* = 10). Significant differences were presented as ** *p* < 0.01 compared to the ND group, ^#^ *p* < 0.05 and ^##^ *p* < 0.01 compared to the HFD group. ND = Normal diet-fed rats treated with vehicle; HFD = High fat diet-fed rats treated with vehicle; EEI100 = HFD-fed rats treated with 100 mg/kg/day of EEI; EEI200 = HFD-fed rats treated with 200 mg/kg/day of EEI; Pio = HFD-fed rats treated with 20 mg/kg/day of pioglitazone.

The skeletal muscle and liver are the main target of glycogen storage, managed by insulin. Besides insulin resistance, impaired glycogen synthesis is another major abnormality in type 2 diabetes and obesity [23,24]. Our study, therefore, verified whether the EEI affects hepatic and muscle glycogen accumulation in HFD-induced obese rats. As shown in Figure 2, the hepatic and soleus muscle glycogen content in HFD-induced obese rats enormously lessened compared with that in the ND group. Interestingly, all doses of EEI and pioglitazone significantly increased liver glycogen content in HFD-fed rats. On the contrary, only 200 mg/kg/day of EEI and pioglitazone significantly increased glycogen content in the soleus muscle of HFD-fed rats. Interestingly, 200 mg/kg/day of EEI had more effective than pioglitazone in increasing the soleus glycogen content in HFD-fed rats. All of these results suggested that EEI treatment abrogated the reduction of glycogen content in the liver comparable to 20 mg/kg/day pioglitazone, while 200 mg/kg/day EEI obviously increased soleus muscle glycogen content 1.3 times over the standard drug, pioglitazone.

### 3.5. EEI Improved Glucose Transporter and Insulin Receptor Substrate Gene Expression in Liver and Soleus Muscle Tissues of HFD-Induced Obese Rats

A family of glucose transporter (GLUTs) is involved in controlling tissue-specific glucose uptake and metabolism in the liver, skeletal muscle, and adipose tissue to maintain blood glucose homeostasis. It is well recognized that GLUT2, the major regulator of hepatic glucose influx [25], and GLUT4, the main modulator for insulin- and contraction-stimulated glucose uptake in skeletal muscle, are the crucial contributors to the control of blood glucose [26,27,28].

**Figure 3:**
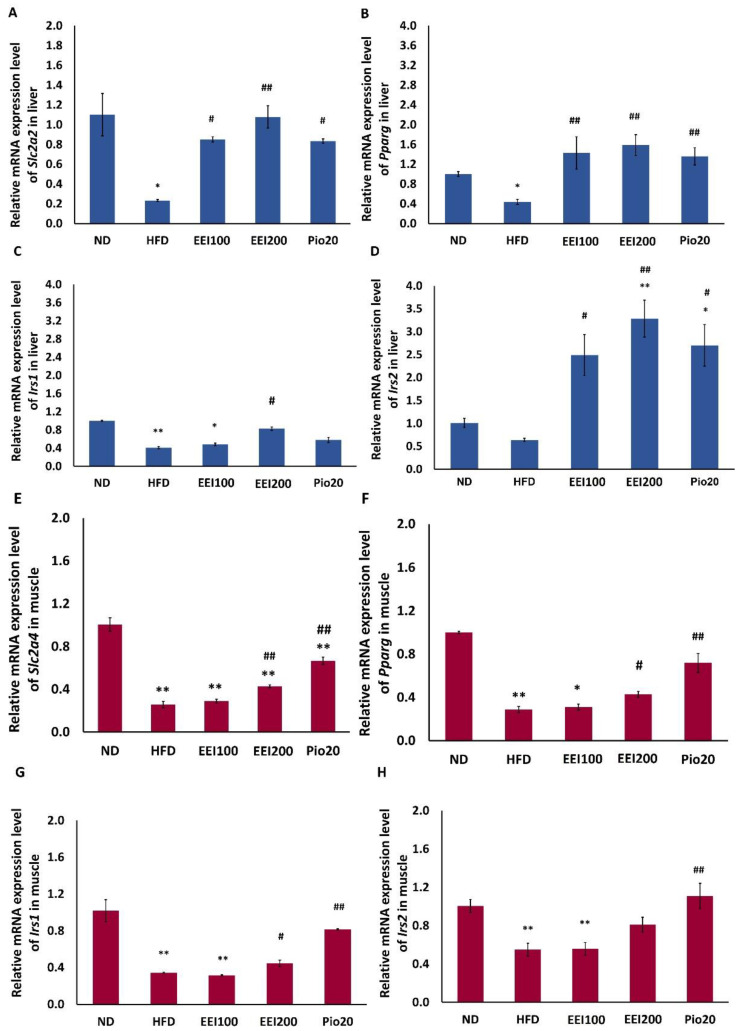
The effect of 2-week feeding EEI on gene expression of *Slc2a2* (**A**), *Pparg* (**B**), *Irs1* (**C**), *Irs2* (**D**) in liver and *Slc2a4* (**E**), *Pparg* (**F**), *Irs1* (**G**), *Irs2* (**H**) in soleus muscle tissues. Results are presented as the means ± SEM (*n* = 10). Significant differences were presented as * *p* < 0.05 and ** *p* < 0.01 compared to the ND group, ^#^ *p* < 0.05 and ^##^ *p* < 0.01 compared to the HFD group. ND = Normal diet-fed rats treated with vehicle; HFD = High fat diet-fed rats treated with vehicle; EEI100 = HFD-fed rats treated with 100 mg/kg/day of EEI; EEI200 = HFD-fed rats treated with 200 mg/kg/day of EEI; Pio = HFD-fed rats treated with 20 mg/kg/day of pioglitazone.

As shown in Figure 3A,E with the sensitive real-time PCR technique, mRNA expression of solute carrier family 2 (facilitated glucose transporter), members 2 and 4 (*Slc2a2/Glut2* and *Slc2a4/Glut4*) in the liver and soleus muscle tissues, respectively, were downregulated in obese rats induced by HFD compared to the normal rats fed by ND. Interestingly, treatment with all doses of EEI and pioglitazone upregulated mRNA expression of *Slc2a2* in the liver of HFD-fed rats. In addition, only 200 mg/kg/day of EEI and pioglitazone increased mRNA expression of *Slc2a4* in the soleus muscle of HFD-fed rats.

We also observed that the expression of insulin receptor substrates 1 (*Irs1*) and 2 (*Irs2)* in liver and soleus muscle tissues was reduced in the HFD group in comparison to the ND group. Interestingly, only 200 mg/kg/day of EEI and pioglitazone considerably increased mRNA expression of *Irs1* in both the liver and soleus muscle of HFD-fed rats. In addition, all doses of EEI and pioglitazone equally increased the mRNA expression of *Irs2* in the liver of HFD-fed rats. However, only pioglitazone increased the mRNA expression of *Irs2* in the soleus muscle of HFD-fed rats (Figure 3C,D,G,H).

Furthermore, the expression of *Pparg* was considerably lower in the liver and soleus muscle tissues of the HFD group than that of the ND group. Interestingly, treatment with all doses of EEI and pioglitazone equally increased the mRNA expression of *Pparg* in the liver of HFD-fed rats. In addition, only 200 mg/kg/day of EEI and pioglitazone increased mRNA expression of *Pparg* in the soleus muscle of HFD-fed rats (Figure 3B,F).

### 3.6. EEI Decreased Liver Weight and Triglyceride in HFD-Induced Obese Rats

**Figure 4:**
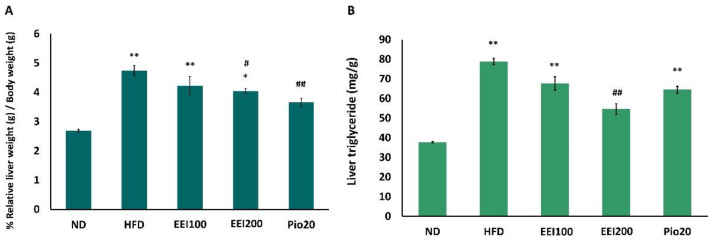
The effect of 2-week feeding EEI on %relative liver weight/body weight (**A**) and liver triglycerides (mg/g) (**B**). Results are presented as the means ± SEM (*n* = 10). Significant differences were presented as * *p* < 0.05 and ** *p* < 0.01 compared to the ND group, ^#^ *p* < 0.05 and ^##^ *p* < 0.01 compared to the HFD group. ND = Normal diet-fed rats treated with vehicle; HFD = High fat diet-fed rats treated with vehicle; EEI100 = HFD-fed rats treated with 100 mg/kg/day of EEI; EEI200 = HFD-fed rats treated with 200 mg/kg/day of EEI; Pio = HFD-fed rats treated with 20 mg/kg/day of pioglitazone.

The liver is one of the body organs that plays a major role in glucose and lipid metabolism. Excessive lipid accumulation in the liver may cause liver dysfunction and hepatic insulin resistance [29,30]. EEI-induced change in the liver of insulin-resistant-obese rats was therefore studied. In comparison to the ND group, the HFD group exhibited a significant increase in %relative liver weight/body weight (*p* < 0.01; Figure 4A). Interestingly, 200 mg/kg/day of EEI and pioglitazone significantly decreased %relative liver weight/body weight in HFD-fed rats (Figure 4A). Moreover, the group fed HFD displayed an apparent increase in liver triglyceride (78.8 mg/g) compared to the ND group (37.7 mg/g; Figure 4B). Conversely, a significant reduction of triglyceride accumulated in the liver after the co-treatment with 200 mg/kg/day of EEI was observed when compared to the group fed HFD only. The results suggest that EEI treatment reversed HFD-induced hepatic steatosis.

### 3.7. EEI Ameliorated the Hepatic Histological Alteration and Fat Deposition in HFD-Induced Obese Rats

**Figure 5:**
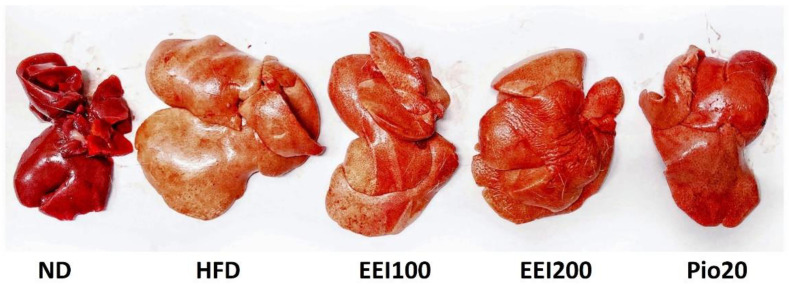
The effect of 2-week feeding EEI on macroscopic liver appearance. ND = Normal diet-fed rats treated with vehicle; HFD = High fat diet-fed rats treated with vehicle; EEI100 = HFD-fed rats treated with 100 mg/kg/day of EEI; EEI200 = HFD-fed rats treated with 200 mg/kg/day of EEI; Pio = HFD-fed rats treated with 20 mg/kg/day of pioglitazone.

**Figure 6:**
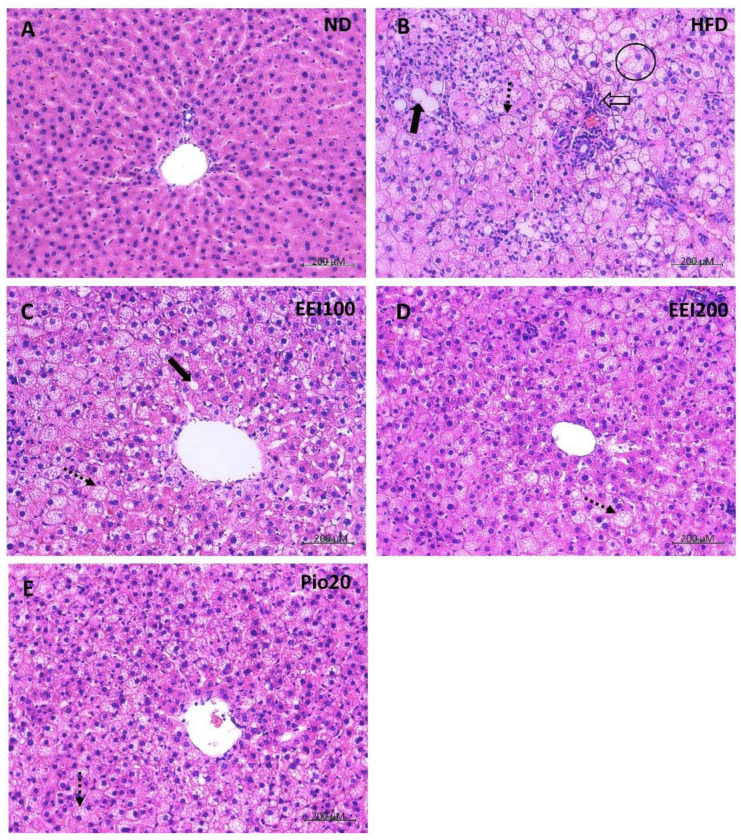
The effect of 2-week feeding EEI on microscopic liver appearance (200x). ND = Normal diet-fed rats treated with vehicle (**A**); HFD = High fat diet-fed rats treated with vehicle (**B**); EEI100 = HFD-fed rats treated with 100 mg/kg/day of EEI (**C**); EEI200 = HFD-fed rats treated with 200 mg/kg/day of EEI (**D**); Pio = HFD-fed rats treated with 20 mg/kg/day of pioglitazone (**E**). Macrovesicular steatosis (bold arrows): large size of lipid droplets are found in hepatocytes. Microvesicular steatosis (dotted line arrow): small size of lipid droplets are presented in hepatocytes. Hypertrophy (within the circle): the representative cells are larger than the surrounding steatotic hepatocytes. The cluster of inflammatory cells (open arrow): Aggregations of inflammatory cells are found in hepatocytes.

After treatment, the liver of the ND-fed rats exhibited dark red color and sharp edge, whereas the liver of HFD-fed rats demonstrated light color and irregular surface suggestive of fatty liver (Figure 5). Notably, treatment with EEI and pioglitazone alleviated hepatic morphological change. The histopathological determination of the liver tissue assisted with hematoxylin and eosin staining paraffin tissue sections, and a modified Kleiner scoring system [22] of the ND group revealed normal lobular architecture (Figure 6A and Table 4). On the contrary, a number of large- and small-sized lipid droplets were markedly observed in the liver of the HFD group, attributed to macrovesicular steatosis and microvesicular steatosis, respectively. Moreover, hepatocellular hypertrophy and inflammation were also visibly found in the liver of the HFD group, indicating NASH (Figure 6B). Interestingly, the liver tissue of HFD-fed rats treated with 100, 200 mg/kg/day of EEI and pioglitazone remarkably decreased macrovesicular steatosis (Figure 6C–E). Additionally, 200 mg/kg/day of EEI administration obviously lessened hepatocellular hypertrophy and cluster of inflammatory cells in HFD-fed rats, whereas pioglitazone could lower only the inflammation.

### 3.8. EEI Altered Hepatic Gene Expression Related to Lipid Metabolism and Inflammation

**Figure 7:**
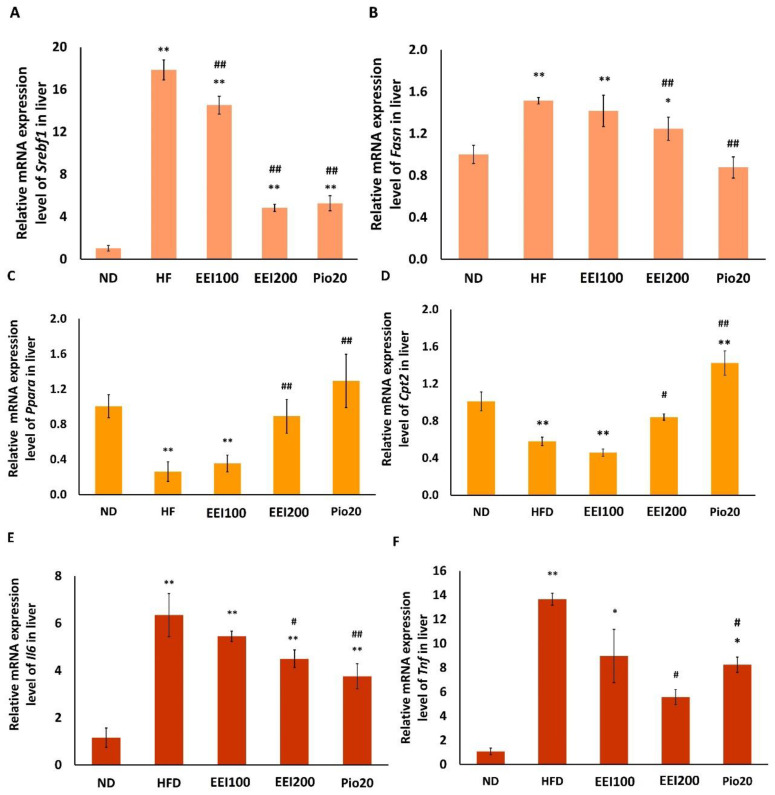
The effect of 2-week feeding EEI on gene expression of *Srebf1* (**A**), *Fasn* (**B**), *Ppara* (**C**), *Cpt2* (**D**), *Il6* (**E**), *Tnf* (**F**) in liver. Results are presented as the means ± SEM (*n* = 10). Significant differences were presented as * *p* < 0.05 and ** *p* < 0.01 compared to the ND group, ^#^ *p* < 0.05 and ^##^ *p* < 0.01 compared to the HFD group. ND = Normal diet-fed rats treated with vehicle; HFD = High fat diet-fed rats treated with vehicle; EEI100 = HFD-fed rats treated with 100 mg/kg/day of EEI; EEI200 = HFD-fed rats treated with 200 mg/kg/day of EEI; Pio = HFD-fed rats treated with 20 mg/kg/day of pioglitazone.

To evaluate the molecular mechanism involved in the inhibitory response of EEI on HFD-induced hepatic steatosis, gene expressions of hepatic lipid metabolism and inflammation in rats fed the ND and the HFD without vs. with EEI treatment were compared. As shown in Figure 7A,B, the HFD greatly increased the lipogenic gene expression of sterol regulatory element binding transcription factor 1 *(Srebf1/SREBP-1c)* and fatty acid synthase *(Fasn)* compared with the ND rats. All doses of EEI and pioglitazone significantly decreased mRNA expression of *Srebf1* in the liver of HFD-fed rats. In addition, only 200 mg/kg/day of EEI and pioglitazone significantly decreased the mRNA expression of *Fasn* in the liver of HFD-fed rats.

Additionally, HFD also dramatically inhibited mRNA expression of fatty acid oxidation-related genes, *Ppara* and carnitine palmitoyltransferase 2 *(Cpt2)*, compared with the ND rats. Administration with 200 mg/kg/day of EEI and pioglitazone significantly increased mRNA expression of *Ppara* and *Cpt2* in the liver of HFD-fed rats.

Furthermore, HFD intensely induced inflammatory-related gene expression of interleukin 6 (*Il6*) and tumor necrosis factor (*Tnf)* in the liver, compared with the ND rats. On the contrary, 200 mg/kg/day of EEI and pioglitazone significantly decreased mRNA expression of both *Il6* and *Tnf* in the liver of HFD-fed rats.

Taken together, the findings suggest that EEI inhibited HFD-induced lipogenesis and inflammatory responses and increased fatty acid oxidation in the liver of rats.

## 4. Discussion

Excess dietary fat intake normally leads to induce obesity and further promotes insulin resistance, and it is well known that insulin resistance frequently leads to the progress of T2DM [31,32,33]. Obesity may also influence fat accumulation in the liver by reducing adiponectin levels, consequently reducing fatty acid oxidation. Insulin resistance is crucial for the progression of NAFLD directly through raising de novo lipogenesis and indirectly through raising free fatty acid flux to the liver [32,33]. It has been documented that insulin resistance is closely associated with NAFLD since approximately 70–80% of obese and diabetic subjects have NAFLD [34,35]. The need for a safe and efficient substance for improving conditions of insulin resistance is still a worthy scientific research concern. Exploring novel medications from natural products is therefore interesting to solve these health problems occurring in diabetic patients. 

The fern *C. terminans* has long been consumed as a vegetable [36], whereas its traditional use for pharmacotherapy is absent. Nonetheless, several biological activities of interruptin derivatives isolated from *C. terminans* have recently been reported in vitro, which included anti-diabetic properties [15,16]. The chief aim of this study was to elucidate whether *C. terminans* extract enriched by interruptins A and B (EEI) could ameliorate insulin resistance and NAFLD in HFD-induced obese rats.

After 12 weeks of an HFD treatment, the obese insulin-resistant rat model was successfully developed, as evidenced by the significant gain in body weight, elevated plasma insulin and HOMA- IR index, hyperglycemia, and impaired glucose tolerance, which are known to exacerbate the progression of type 2 diabetes. Additionally, long-term HFD intervention in rats efficiently induced NASH as demonstrated by hepatic steatosis and inflammation, together with the increase in serum AST, ALT, and ALP levels and inflammatory-related gene expression of *Il6* and *Tnf*.

The HFD-fed rats that had received EEI exhibited lower glucose, insulin levels, and HOMA- IR index than the control group. It also decreased glycemic responses after challenges with glucose (2 g/body weight). The EEI improved glucose metabolism in HFD-fed rats through the up-regulation of *Slc2a4* and *Scl2a2* gene expression in soleus muscle and liver cells, respectively. Stimulation of insulin signaling pathways through the solute carrier family 2 (facilitated glucose transporter) members 4 and 2 was the mechanism underlying the beneficial effects of EEI on obesity-induced insulin resistance.

Furthermore, suppression of the insulin receptor signaling pathway by HFD-induced obesity or inflammation is a vital mechanism of insulin resistance. Insulin receptor signaling deficiency is mostly reflected in insulin resistance [37]. To investigate insulin signaling, gene expression of *Irs1* and *Irs2* in the liver and soleus muscle cells was also examined. *Irs1* and *Irs2* expressions were activated in HFD + EEI treated groups compared to the HFD group. Of note, the expression of *Irs2* in the muscle tends to increase modestly. Additionally, it is well established that insulin resistance and hepatosteatosis lead to reduced glycogen synthesis [29,38] and that enhanced hepatic glycogen synthesis recovers glucose intolerance [39]. The results of the present study exhibited that EEI increased hepatic and soleus muscle glycogen synthesis in HFD-induced obese rats. The results support evidence that EEI may improve hyperglycemia in type 2 diabetes.

Excessive accumulation of visceral fat is a general stimulator of obesity-related comorbidities [40,41]. Visceral adipose tissue is well recognized to be more correlated to the development of metabolic syndromes [42]. It secrets inflammation cytokine, which is one of the key factors to induce insulin resistance [43]. It has been documented that body weight reduction is a crucial way suggested for type 2 diabetes and NAFLD patients since weight loss could decrease hepatic triglyceride accumulation with an improvement of insulin sensitivity [44]. EEI intervention markedly prohibited the body weight gain of HFD-fed rats compared with the rats in the HFD group, suggesting that the anti-obesity effect of EEI may lessen the progression of type 2 diabetes and NAFLD.

The administration of EEI obviously protected against liver weight gain and reduced hepatic fat accumulation induced by HFD. Furthermore, EEI treatment considerably improved hepatic function, indicated by AST and ALT levels in HFD-fed rats. It has been reported that the increased serum AST and ALT levels are primary anomalies found in NAFLD and other hepatic disease patients [45]. The histopathological analysis presented that EEI also reduced the HFD-induced fatty change and inflammation in the liver, which indicates that EEI could protect against HFD-induced NAFLD, which included hepatic steatosis and NASH. We proposed that the effects of EEI were modulated through the regulation of lipogenesis, fatty acid oxidation, and anti-inflammation.

Fatty acid synthase (FAS)1, an essential enzyme in the lipogenesis pathway, catalyzes all of the steps in the conversion of malonyl-CoA to palmitate. *Fasn* gene expression is mainly controlled at the transcription level and is affected by both hormonal and nutritional signals [46,47]. SREBP-1c is a transcription factor controlling the expression of lipogenesis-associated enzymes and fatty acid desaturation and is responded to by insulin. SREBP-1c is involved in the progress of hepatic steatosis [48]. The inhibitory effect on *Srebf1* gene expression was approved in the rats treated with EEI, and this was, in turn, expected to downregulate the expression of *Fasn* lipogenic genes. These findings indicated that the hepatic lipid-reducing effect of EEI in HFD-fed rats might be due to its inhibitory activity on hepatic lipogenesis.

Additionally, we found that EEI could up-regulate *Ppara* and *Cpt2* gene expression in HFD-fed rats. PPARA regulates fatty acid oxidation that occurs in the mitochondria and gives a source of energy to produce ATP. However, the entry of fatty acid across the mitochondrial membrane is activated by PPRE-dependent regulation of CPT1 and CPT2, which are situated in the outer and inner mitochondrial membrane, respectively [49,50,51,52]. Moreover, it has been documented that PPARA exhibits anti-inflammatory properties [53]. Its stimulation can cause inhibition of nuclear factor (NF) kB activation and inflammatory gene expression [53,54,55]. Hence, PPARA activation performs an important role in the alteration of hepatic steatosis and steatohepatitis because of its effects on the facilitation of the fatty acid oxidation system and downregulation of inflammatory response [56,57,58].

Interleukin 6 (IL6) and tumor necrosis factor (TNF) are conventional pro-inflammatory cytokines believed to be risk factors for diabetes progression due to their disturbance of insulin signaling [59,60]. TNF is mostly generated by activated macrophages, whereas IL6 can be produced in a lot of immune cells involving activated macrophages and lymphocytes [61]. Besides macrophages, the inflammatory cytokines IL6 and TNF are additionally emitted from fat tissues and are known to be increased in obesity and insulin-resistant patients [62,63,64]. Moreover, it has been revealed that these two inflammatory cytokine levels are elevated in the plasma of NAFLD and NASH patients [65,66,67]. In this study, the diminished effect of EEI on inflammatory gene expression in liver tissue of HFD-induced insulin resistance and NAFLD rats was clearly observed from the decrease of *Tnf* and *Il6*. These suggested that activation of fatty acid oxidation and anti-inflammatory responses by EEI may be involved in the attenuation of insulin resistance and NAFLD in HFD-induced obese rats.

In light of these findings, it appears promising that the beneficial pharmacological outcomes from this study were probably due to active interruptin derivatives carried in EEI. Since EEI was enriched in interruptins A and B that demonstrate anti-diabetes, anti-inflammatory, and anti-oxidative stress [13,14,15,16], it has been revealed that interruptins A and B effectively promoted glucose consumption to hepatocytes and adipocytes via modulation of the PPAR pathway combined with upregulation of the *Slc2a1/Glut1* gene and GLUT2 and GLUT4 proteins [15,16]. Additionally, they were found to stimulate hepatic glycogen accumulation facilitated by both PPARA and PPARG proteins [16]. Indeed, interruptin B was found to act as a PPARA and PPARG agonist [15]. Moreover, interruptin A shares a similar core structure with interruptin B and was also predicted to be a dual PPARA and PPARG ligand (unpublished data). PPARG is a nuclear receptor that normally participates in both lipid and glucose homeostasis [68]. It is well established that treatment with TZD, a PPARG agonist in T2DM, leads to PPARG activation and causes enhanced insulin sensitivity, resulting in decreased plasma insulin and glucose levels [69]. PPARA is another transcriptional regulator involved in fatty acid catabolism and also activates fatty acid oxidation [70]. In addition, dyslipidemia is considered by raised circulating triglyceride levels in combination with declined HDL levels [71]. On the other hand, PPARA agonists are capable of decreasing plasma triglycerides and increasing HDL levels [72]. As a result, we observed that the levels of serum HDL in EEI-treated HFD-fed rats were considerably elevated as compared with the HFD group. This beneficial effect was plausible due to PPARα activation by interruptins A and B in EEI. Our findings in this study showed that EEI could up-regulate the liver and muscle gene expression of both *Ppara* and *Pparg,* which significantly attenuate insulin resistance and NAFLD in HFD-induced obese rats and these effects are largely due to the presence of interruptins A and B in EEI.

## 5. Conclusions

The results of this study indicated that EEI from *C. terminans* is effective in anti-diabetic and associated metabolic disease NAFLD against HFD-induced obesity. EEI reverses insulin resistance by decreasing insulin and glucose levels, HOMA-IR index, and improving impaired glucose tolerance. The beneficial effect of EEI on insulin resistance and diabetes is mediated by the insulin signaling pathway and inflammatory response. EEI also attenuates NASH, which is a progressive form of NAFLD, by reducing lipid accumulation, fatty change, inflammation and increase of AST and ALT enzymes in the liver of rats. The potential of EEI on NASH remedy is associated with the regulation of lipogenesis, fatty acid oxidation, and anti-inflammation. *C. terminans* extract enriched by in interruptins A and B, therefore, has strong prospects to be developed as a natural health product for the management of insulin resistance, type 2 diabetes, and NAFLD in patients.

## Figures and Tables

**Table 1:** List of primers for real-time PCR analysis.

Genes	Forward Primer (5′-3′)	Gene Location	NCBI Reference Sequence	Size (bp)
*Irs1*	F: 5′ GCCAATCTTCATCCAGTTGC 3′	1374–1393	NM_012969.2	336
	R: 5′ CATCGTGAGAAGGCATAGG 3′	1710–1691		
*Irs2*	F: 5′ AGTAAACGGAGGTGGCTACA 3′	2142–2161	NM_001168633.1	197
	R: 5′ AAGCTGCTGGAAGTCAGGT 3′	2338–2319		
*Slc2a2*	F: 5′ ATTCGCCTGGATGATTACG 3′	1511–1530	XM_006232207.3	203
	R: 5′ AGTCCGCCAATGTACTGGAAG 3′	1713–1694		
*Slc2a4*	F: 5′ ATTGGCATTCTGGTTGCCCA 3′	644–663	XM_006246596.4	163
	R: 5′ GGTTCCGGATGATGTAGAGGTA 3′	806–785		
*Srebf1*	F: 5′ GATGCCAACCAGATTCCCTAAG 3′	7431–7452	NM_001276708.1	210
	R: 5′ TCAGTTGTTTCTTTGCCTTCCA 3′	7640–7619		
*Cpt2*	F: 5′ GTGGCAAGGAGTTCCTGAAG 3′	1494–1513	XM_039109299.1	411
	R: 5′ TGGTTCATCTGCTGGTATGC 3′	1904–1885		
*Fasn*	F: 5′ TCCCAGGTCTTGCCGTGC 3′	6224–6241	NM_017332.2	260
	R: 5′ GCGGATGCCTAGGATGTGTGC 3′	6483–6463		
*Tnf*	F: 5′ GTAGCCCACGTCGTAGCAAA 3′	455–474	HQ201305	347
	R: 5′ CCCTTCTCCAGCTGGAAGAC 3′	801–782		
*Il6*	F: 5′ AGTTGCCTTCTTGGGACTGA 3′	97–116	NM_012589.2	485
	R: 5′ GAGCATTGGAAGTTGGGGTA 3′	581–562		
*Ppara*	F: 5′ GATTCGGAAACTGCAGACCTC 3′	868–888	XM_017594680.1	444
	R: 5′ TAGGAACTCTCGGGTGATGA 3′	1311–1292		
*Pparg*	F: 5′ CAAAGTAGAACCTGCATCTCC 3′	402–422	XM_006237009.3	156
	R: 5′ CCTTCACAAGCATGAACTCC 3′	557–538		
*Act*	F: 5′ GTACCACTGGCATTGTGATG 3′	518–537	NM_031144.3	541
	R: 5′ ATCTTCATGGTGCTAGGAGC 3′	1058–1039		

**Table 2:** The effect of 2-week feeding EEI on blood glucose, insulin, HOMA-IR, and OGTT of rats.

Parameters	ND	High Fat Diet			
		HFD	EEI100 (mg/kg/day)	EEI200 (mg/kg/day)	Pio20 (mg/kg)
Blood glucose (mg/dL)	126.8 ± 2.9	139.4 ± 4.3 **	128.3 ± 2.1 ^#^	126.1 ± 2.9 ^##^	124.1 ± 2.3 ^##^
Insulin (ng/mL)	2.8 ± 0.6	5.2 ± 0.4 **	4.4 ± 0.8 *	3.2 ± 0.4 ^#^	3.5 ± 0.1 ^#^
HOMA-IR	21.8 ± 4.3	43.8 ± 2.2 **	34.6 ± 6.6 *	24.2 ± 2.5 ^##^	26.8 ± 0.7 ^#^
OGTT AUC (mg/dL × min × 10^4^)	4.8 ± 0.1	5.6 ± 0.2 **	5.1 ± 0.2	5.0 ± 0.1 *^,##^	5.0 ± 0.1 ^##^

Results are presented as the means ± SEM (*n* = 10). Significant differences were presented as * *p* < 0.05 and ** *p* < 0.01 compared to the ND group, ^#^ *p* < 0.05, and ^##^ *p* < 0.05 compared to the HFD group. HOMA-IR: the homeostasis model assessment of insulin resistance, OGTT: oral glucose tolerance test, ND = Normal diet-fed rats treated with vehicle; HFD = High fat diet-fed rats treated with vehicle; EEI100 = HFD-fed rats treated with 100 mg/kg/day of EEI; EEI200 = HFD-fed rats treated with 200 mg/kg/day of EEI; Pio = HFD-fed rats treated with 20 mg/kg/day of pioglitazone.

**Table 3:** The effect of 2-week feeding EEI on biochemical parameters.

Parameters	ND	High Fat Diet			
		HFD	EEI100 (mg/kg/day)	EEI200 (mg/kg/day)	Pio20 (mg/kg/day)
Triglyceride (mg/dL)	86.6 ± 14.8	82.1 ± 5.7	77.0 ± 4.0	75.3 ± 4.5	57.3 ± 6.1 **^,##^
Cholesterol (mg/dL)	88.6 ± 3.3	114.7 ± 3.8	107.2 ± 2.8 **	106.2 ± 3.9 **	101.7 ± 7.0 *^,#^
LDL (mg/dL)	33.0 ± 2.9	69.7 ± 3.3 **	61.5 ± 2.6 **	61.0 ± 3.2 **	69.9 ± 5.8 **
HDL (mg/dL)	29.3 ± 1.4	25.3 ± 1.5 *	28.0 ± 0.8	30.6 ± 0.8 ^##^	28.0 ± 1.3
AST (U/L)	109.3 ± 15.6	267.8 ± 44.2 **	201.8 ± 16.4 **	196.3 ± 17.4 **^,#^	194.5 ± 15.8 **^,#^
ALT (U/L)	39.1 ± 3.7	157.0 ± 18.0 **	128.6 ± 17.8 **	95.7 ± 14.9 **^,##^	112.7 ± 9.8 **^,#^
ALP (U/L)	77.1 ± 1.7	191.3 ± 17.5 **	162.9 ± 11.9 **	151.0 ± 16.3 **	144.9 ± 10.4 *

Results are presented as the means ± SEM (*n* = 10). Significant differences were presented as * *p* < 0.05 and ** *p* < 0.01 compared to the ND group, ^#^ *p* < 0.05, and ^##^ *p* < 0.05 compared to the HFD group. LDL: low-density lipoprotein, HDL: high-density lipoprotein, AST: aspartate aminotransferase, ALT: alanine aminotransferase, ALP: alkaline phosphatase, ND = Normal diet-fed rats treated with vehicle; HFD = High fat diet-fed rats treated with vehicle; EEI100 = HFD-fed rats treated with 100 mg/kg/day of EEI; EEI200 = HFD-fed rats treated with 200 mg/kg/day of EEI; Pio = HFD-fed rats treated with 20 mg/kg/day of pioglitazone.

**Table 4:** The effect of 2-week feeding EEI on the histopathological score of liver tissue.

Histological Features (Score)	ND	High Fat Diet			
		HFD	EEI100 (mg/kg/day)	EEI200 (mg/kg/day)	Pio20 (mg/kg/day)
**Steatosis**					
Microvesicular steatosis (0–3)	0.1 ± 0.1	2.8 ± 0.1 **	2.3 ± 0.2	2.2 ± 0.1	2.3 ± 0.2 *
Macrovesicular steatosis (0–3)	0.1 ± 0.1	2.1 ± 0.1 **	1.2 ± 0.1 **^,#^	1.0 ± 0.1 *^,##^	1.1 ± 0.1 *^,#^
Hypertrophy (0–3)	0.0 ± 0.0	3.0 ± 0.0 **	2.3 ± 0.5 **	1.2 ± 0.4 ^##^	2.0 ± 0.5 **
**Inflammation**					
Number of inflammatory foci/field (0–3)	0.8 ± 0.1	2.3 ± 0.2 **	1.4 ± 0.3	1.3 ± 0.2 ^#^	1.3 ± 0.2 ^#^

The data are presented as mean ± standard error of the mean (SEM). The abnormal distribution data were analyzed using the Kruskal–Wallis test, followed by the Dunn–Bonferroni post hoc test to determine the difference between groups. Statistical significance presented at *^, #^ *p* < 0.05 and **^, ##^ *p* < 0.01 levels.

## Data Availability

All data generated or analyzed during this study are included in this published article.
